# Estimating flight trajectories of breaking balls from four-seam fastballs

**DOI:** 10.3389/fspor.2023.1092520

**Published:** 2023-04-05

**Authors:** Arata Kimura, Hirotaka Nakashima, Shuntaro Kuroyanagi, Yuka Ando, Penhao Liao, Shinji Sakurai

**Affiliations:** ^1^ Department of Sport Science and Research, Japan Institute of Sports Sciences, Kita-ku, Japan; ^2^ Graduate School of Health and Sport Sciences, Chukyo University, Toyota, Japan; ^3^ Research Institute of Health and Sport Sciences, Chukyo University, Toyota, Japan; ^4^ School of Health and Sport Sciences, Chukyo University, Toyota, Japan

**Keywords:** baseball pitching, ball deviation, curveball, four-seam fastball, slider

## Abstract

It is widely acknowledged that understanding the physical mechanics of the flight trajectories of four-seam fastballs and breaking balls is crucial for players and coaches to enhance pitching performance. The characteristics of the flight trajectories of four-seam fastballs and breaking balls have been revealed; however, the relationship between them has not been examined. Here, we show the characteristics of the flight trajectory of breaking balls from the four-seam fastballs. We found that the direction of the deviation of the curveballs could be generally predicted from that of the four-seam fastballs. We also found that the limits of the deviation of the sliders can be determined from the direction of the deviation of the four-seam fastball. This study revealed the deviation of the breaking ball from the four-seam fastballs, which clearly showed the differences in the characteristics between curveballs and sliders. This study moved forward with the description of the physical properties of each pitch type and allowed us to obtain valuable insights and practical implications.

## 1. Introduction

The goal of a pitcher is to overcome the opposing batter during baseball pitching. Pitchers attempt to make it difficult for batters to contact the ball using four-seam fastballs and various breaking balls. Throwing various pitches is especially true for professional players who throw balls with unique flight trajectories. Recently, interest in flight trajectories has increased with the advent of Trackman and PITCHf/x, making it easier to measure flight trajectories (1–3). It is now widely recognized that understanding flight trajectories is important for players and coaches to improve pitching performance.

It was assumed that the four-seam fastballs would follow a straight trajectory (4). However, several studies have shown that the flight trajectory of four-seam fastballs deviates from a straight trajectory (5–7). The flight trajectory in four-seam fastballs during right-hand overhand throwing involves deviations in the upward and the third base direction compared to a free-fall trajectory without consideration of ball rotation. This is because the four-seam fastballs have a backspin and a sidespin. Several previous studies also showed that the ratios of vertical to left-right deviations in four-seam fastballs depend on the pitcher (5–7). This means that some pitchers throw four-seam fastballs with a large left-right deviation and with a small upward deviation, while others throw four-seam fastballs with a small left-right deviation and large upward deviation.

The flight trajectory of a breaking ball is known to be different from that of four-seam fastballs. The flight trajectory of curveballs involves deviations in the downward and first base directions during right-hand overhand throwing compared to a free-fall trajectory (5–7). This is because the pitcher imparts a sidespin in the opposite direction to the four-seam fastballs and a topspin to the ball. Similar to four-seam fastballs, these previous studies have shown that the ratio of vertical to left-right deviations in the curveballs also depends on the pitcher (5–7).

As shown above, previous studies have provided information on the flight trajectories of four-seam fastballs and breaking balls. The question remains: what type of relationship exists between the flight trajectories of four-seam fastballs and breaking balls? The flight trajectory of the breaking balls is empirically expected to depend on that of the four-seam fastballs, therefore, it is hypothesized that there is some relationship between the flight trajectories of four-seam fastballs and breaking balls. However, this relationship has not been examined. Examining this issue will provide a further understanding of the strategies to overcome the batter.

Therefore, the purpose of this study was to examine the relationship between the flight trajectories of four-seam fastballs and breaking balls. To meet this purpose, we determined the correlations between the ratio of vertical to left-right deviations in four-seam fastballs and breaking balls (curveballs and sliders). Furthermore, we determined the relative deviation of the breaking balls (curveballs and sliders) from four-seam fastballs.

## 2. Methods

### 2.1. Participants

Forty-three male amateur baseball pitchers (18 high schoolers, 21 collegiates, and four semi-professionals) participated in this study. The means and standard deviations for height, body mass, and age were 177.4 ± 5.2 cm, 77.4 ± 6.2 kg, and 18.8 ± 2.8 years, respectively. They included overhand, three-quarter, and sidearm throwing pitchers. Twenty-six pitchers were right-handed, and the others were left-handed. The Ethics Committee of Chukyo University approved the study protocol. Before the experiment, the participants were informed of the purpose and experimental protocols of this study. Written informed consent was obtained from all the participants. Informed consent was obtained from the guardians of the minors.

### 2.2. Experimental procedure

The experiment was conducted in outdoor bullpens, which are practice areas for baseball pitching. After a sufficient warm-up, the participants were asked to throw all pitch types that they normally throw in a game, from the pitcher's mound to a catcher. The catcher was 1 m away from home plate and there was no hitter in the batter's box. The experiment continued until one satisfactory pitch was obtained for each pitch type, as judged by the participants.

### 2.3. Data collection

The speed of the pitched ball was measured using a radar gun (Stalker SPORTS-2, Applied Concepts Inc., Dallas, TX, USA) placed behind the catcher. To determine the angular velocity of the pitched ball, a high-speed video camera (MEMERECAM MX, NAC Image Technology Inc., Japan) was placed approximately 1 m behind the pitcher to record the ball with approximately 100 small marks on the surface. The camera was adjusted to be level with each pitcher's release height. The optical axis of the camera lens was directed in the home plate direction. The camera was set at a frame rate of 1,000 fps and an exposure time of 1/2000–1/5000 s, depending on the brightness.

### 2.4. Data processing

Among the pitch types, we focused only on four-seam fastballs, curveballs, and sliders in this study. This is because while many participants pitched these pitches, few participants pitched the other types, such as changeup. Each pitcher threw a four-seam fastball, 32 pitchers threw a curveball, and 33 pitchers threw a slider.

Motion analysis software (Frame DIAS V, Q'sfix Co., Japan) was used to obtain the positional coordinates on the screen from the video images recorded by the camera. The top, bottom, left, and right edges of the balls and small marks immediately after ball release were manually digitized. Before the analysis, lateral inversion processing was performed on the data of the left-handed pitchers to integrate the data of the left- and right-handed pitchers. The X, Y, and Z axes of the global coordinate system define the right-left, forward-backward, and upward-downward directions, respectively. The positive values of the X axis are in the right direction, those of the Y axis are in the forward direction, and those of the Z axis are in the upward direction. The origin of the global coordinate system is the center of the pitcher's plate. The angular velocity of the pitched ball was determined by using the method proposed by Jinji and Sakurai (5). The angular velocity was resolved into a back/top spin component (***ω_x_***), a gyro spin component (***ω_y_***), and a side spin component (***ω_z_***). The ***ω_x_***, ***ω_y_***, and ***ω_z_*** are the angular velocities about the X, Y, and Z axes of the global coordinate system, respectively. The positive values of the top/backspin (***ω_x_***) indicate the backspin. The positive values of the gyro spin (***ω_y_***) indicate that the ball rotates clockwise about the Y-axis from the pitcher's perspective. The positive values of the sidespin (***ω_z_***) indicate that the ball rotates counterclockwise about the Z-axis viewed from above.

We calculated the deviation from release location to home plate for each pitch. The deviation was calculated as the differences between the coordinates at the arrival position of the ball with and without considering ball rotation. The flight trajectory and arrival position of a ball are obtained using an equation of motion, given the positions (***x***_***_0_***, ***y***_***_0_***, ***z***_***_0_***), translational velocities (***v_x_***_***_0_***, ***v_y_***_***_0_***, ***v_z_***_***_0_***), and angular velocities (***ω_x_***_***_0_***, ***ω_y_***_***_0_***, ***ω_z_***_***_0_***) at ball release (8). The ***x***_***_0_*** and ***y***_***_0_*** were set to 0, and ***z***_***_0_*** was set to the average height of the participants. The ***v_x_0_*** and ***v_z_0_*** were set to 0, and ***v_y_0_*** was set to the value obtained using the speed gun. The angular velocities at ball release (***ω_x_0_***, ***ω_y_0_***, ***ω_z_0_***) were set to the values obtained from actual measurements. The equation of motion used in this study is given by:

(1) $$\;ma\;=F_{d}+F_{l}+mg$$

where *m* (=0.139 kg) is the ball mass, ***a*** is the translational acceleration of the ball, ***F_d_*** is the drag force, ***F_l_*** is the lift force, and ***g*** (=9.81 m/s^2^) is the gravitational acceleration. The drag (***F_d_***) and lift (***F_l_***) forces are expressed as:

(2) $$\;F_{d}=-\frac{1}{2}\;\rho \;C_{d}\;A\;V\;V$$

(3) $$\;F_{l}\;=\;\frac{1}{2}\;\rho \;\;C_{l}\;A\;V^{2}\;\frac{\omega \times V}{\left|\omega \times V\right|}$$

where *ρ* is the fluid density of the air, *A* is the cross-sectional area of the ball, *V* is the ball speed, ***V*** is the translational velocity of the ball, and ***ω*** is the angular velocity of the ball. *C_d_* and *C_l_* are the drag and lift coefficients, respectively. *C_d_* depends on the Reynolds number Re = *V*d/*ν* (d: ball diameter, *ν*: kinematic viscosity of air) and was determined using the method of a previous study (9). We used the *C_d_* calculated by this method because the *C_d_* is time-varying and includes the effect of drag crisis, whereas the *C_d_* used in previous studies is generally a constant value and does not consider drag crisis (2, 10). *C_l_* strongly depends on the spin parameter *S* = *r**ω***/|**V**| (*r*: ball radius) and was determined using the method developed in previous studies (10, 11).

The rotation of the ball is decayed by the aerodynamic moment, and the rotation decay is given by:

(4) $$\;I\frac{d\omega }{dt}\;=M$$

where *I* (=2/5 *mr^2^*) is the moment of inertia of the ball, and ***M*** is the moment on the ball. The moment on the ball (***M***) is expressed as follows:

(5) $$\;M=\frac{1}{2}\;\rho \;C_{m}\;A\;V^{2}\;\;d\frac{\omega }{\left|\omega \right|}$$

where *C_m_* denotes the aerodynamic moment coefficient. *C_m_* is a function of the spin parameter and was determined using the method of a previous study (12).

These equations indicate that the lift force (***F_l_***) and moment on the ball (***M***) include an angular velocity term. Therefore, the arrival position with and without consideration of the ball rotation can be calculated depending on the presence or absence of the lift force (***F_l_***) and the moment on the ball (***M***).

The fourth-order Runge–Kutta method was used to numerically integrate Equation (1), thereby obtaining the position coordinates of the ball. The time-step size was set to 0.001 s, and the arrival position of the ball was set to 17 m from the ball release.

**Figure 1:**
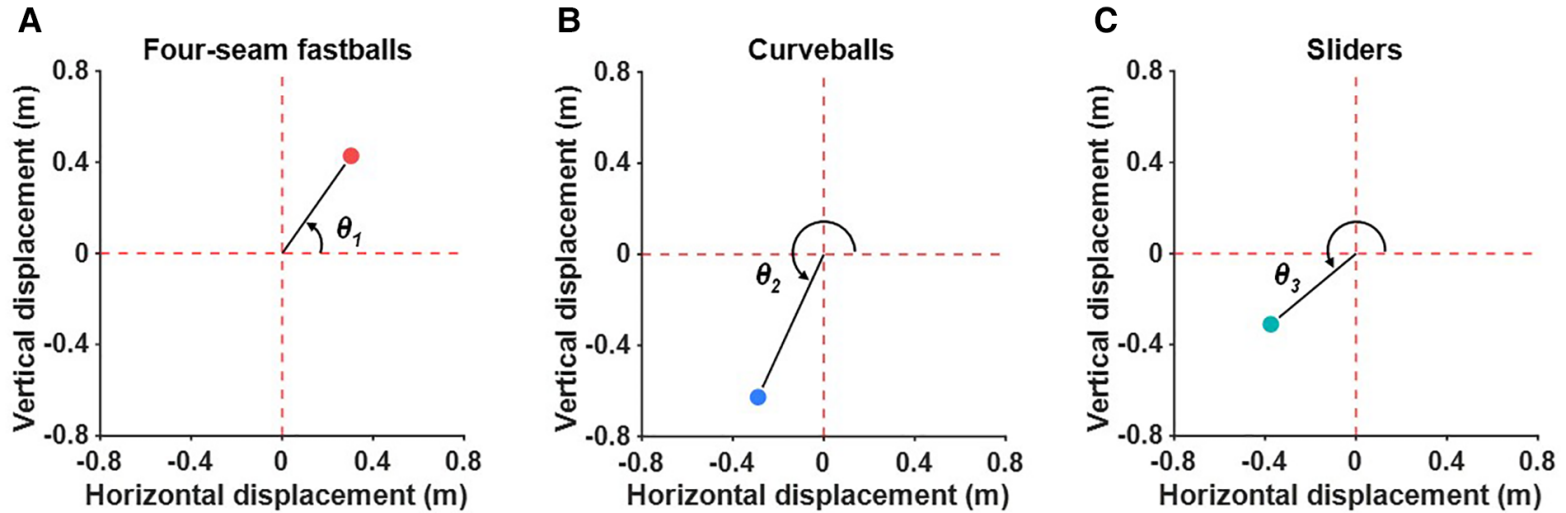
Definition of the angle of each pitch type in the horizontal and vertical displacement plane. The angle between the horizontal displacement axis and the line connecting the origin and the deviation of the four-seam fastballs is defined as *θ_1_* (**A**). *θ_2_* and *θ_3_* are defined as the angle between the horizontal displacement axis and the line connecting the origin and curveballs (**B**) and sliders (**C**), respectively.

The angle of each pitch in the horizontal and vertical displacement plane was calculated to quantify the ratio of vertical to left-right deviations in four-seam fastballs, curveballs, and sliders. *θ_1_* is defined as the angle between the horizontal displacement axis and the line connecting the origin and the deviation of four-seam fastballs (Figure 1A). *θ_2_* and *θ_3_* are defined as the angles between the horizontal displacement axis and the line connecting the origin and the deviation of the curveballs and sliders, respectively (Figures 1B,C). If the angle of the four-seam fastballs (*θ_1_*) is 45°, the amount of deviation of the vertical and left-right directions are equal.

**Figure 2:**
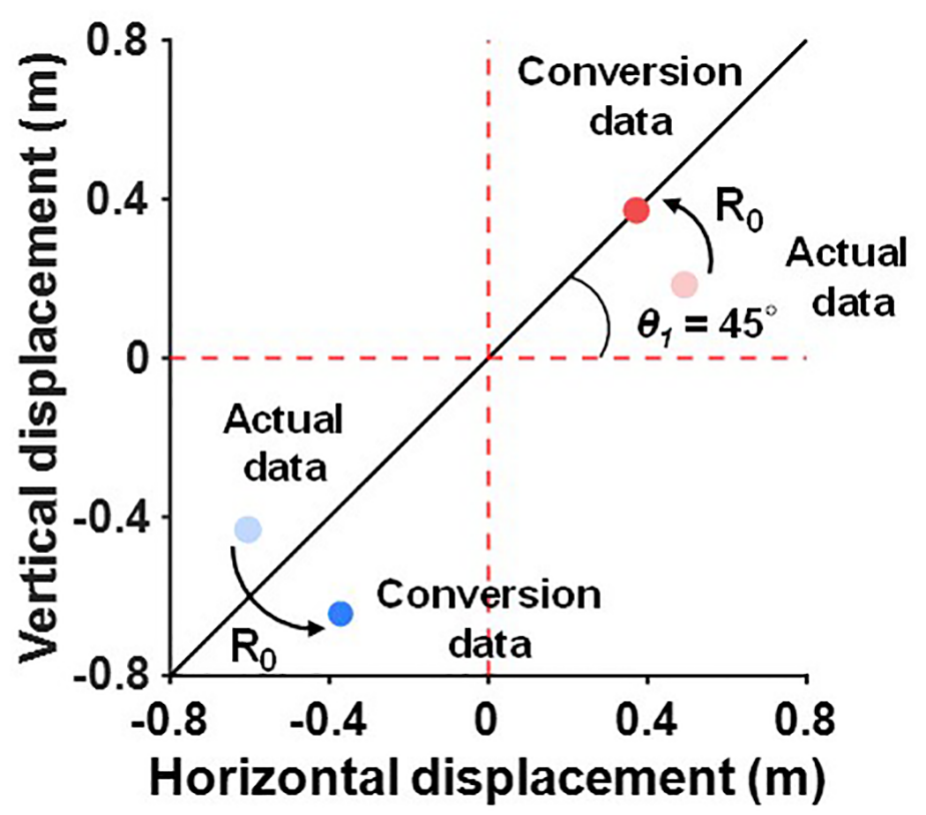
Definition of the relative deviation of breaking balls from the four-seam fastballs. The relative deviation of breaking balls is calculated by the following procedure. First, obtain the rotation matrix (R_0_), which transforms *θ_1_* to 45° in the horizontal and vertical displacement plane. Then, apply the rotation matrix (R_0_) to the deviation of the curveballs and sliders. The transparent red and blue dots indicate the deviation of the four-seam fastball and curveball for actual data. The filled red and blue dots indicate the deviation of the four-seam fastball and curveball after conversion.

We calculated the relative deviation of the curveballs and sliders from the four-seam fastballs. The relative deviation of the curveballs and sliders can be obtained by first determining the rotation matrix (R_0_), which transforms *θ_1_* to 45° in the horizontal and vertical displacement plane (Figure 2). Then, apply the rotation matrix (R_0_) to the deviation of the curveballs and sliders (Figure 2).

### 2.5. Statistics

To evaluate the relationship between the ratio of vertical to left-right deviations in four-seam fastballs and breaking balls, the correlations between the angle of the four-seam fastballs (*θ_1_*) and the angle of the curveballs (*θ_2_*) and between the angle of the four-seam fastballs (*θ_1_*) and the angle of the curveballs (*θ_3_*) were quantified. Because the values are circular, we used circular statistics instead of Pearson's correlation analysis (13). For example, 0 degrees and 360 degrees are the same angles, so a special statistical method, circular statistics, is required for the analysis of angular data. The alpha level was set at 0.05.

## 3. Results

Table 1 lists the ball speed and angular velocity for each pitch type. The four-seam fastballs had a backspin, whereas the curveballs had a topspin. The sliders generally had a topspin but sometimes had a backspin. The gyro spin is generally positive for all pitch types. Sidespin for four-seam fastballs had negative values, whereas sidespin for curveballs and sliders was generally positive.

**Table 1:** The ball speed and angular velocity of each pitch type. The positive values of the top/backspin (***ω_x_***) indicate the backspin. The positive values of the gyro spin (***ω_y_***) indicate that the ball rotates clockwise about the Y-axis from the pitcher's perspective. The positive values of the sidespin (***ω_z_***) indicate that the ball rotates counterclockwise about the Z-axis viewed from above.

Pitch type			Four-seam fastballs	Curveballs	Sliders
*n*			43	32	33
Speed	[m/s]	Mean	36.4	28.3	31.8
		SD	2.0	1.8	2.0
		Min	31.9	25.0	27.3
		Max	40.1	32.3	35.3
Back/top spin (***ω_x_***)	[rad/s]	Mean	150.7	−137.2	−48.9
		SD	31.0	39.1	51.7
		Min	69.0	−200.5	−127.4
		Max	211.3	−48.4	58.4
Gyro spin (***ω_y_***)	[rad/s]	Mean	100.2	111.3	190.6
		SD	39.3	52.2	33.7
		Min	−12.8	2.8	113.9
		Max	166.1	199.4	250.6
Side spin (***ω_z_***)	[rad/s]	Mean	−90.0	84.8	76.4
		SD	44.1	34.9	41.1
		Min	−182.0	25.9	−24.7
		Max	9.2	198.9	179.1

**Figure 3:**
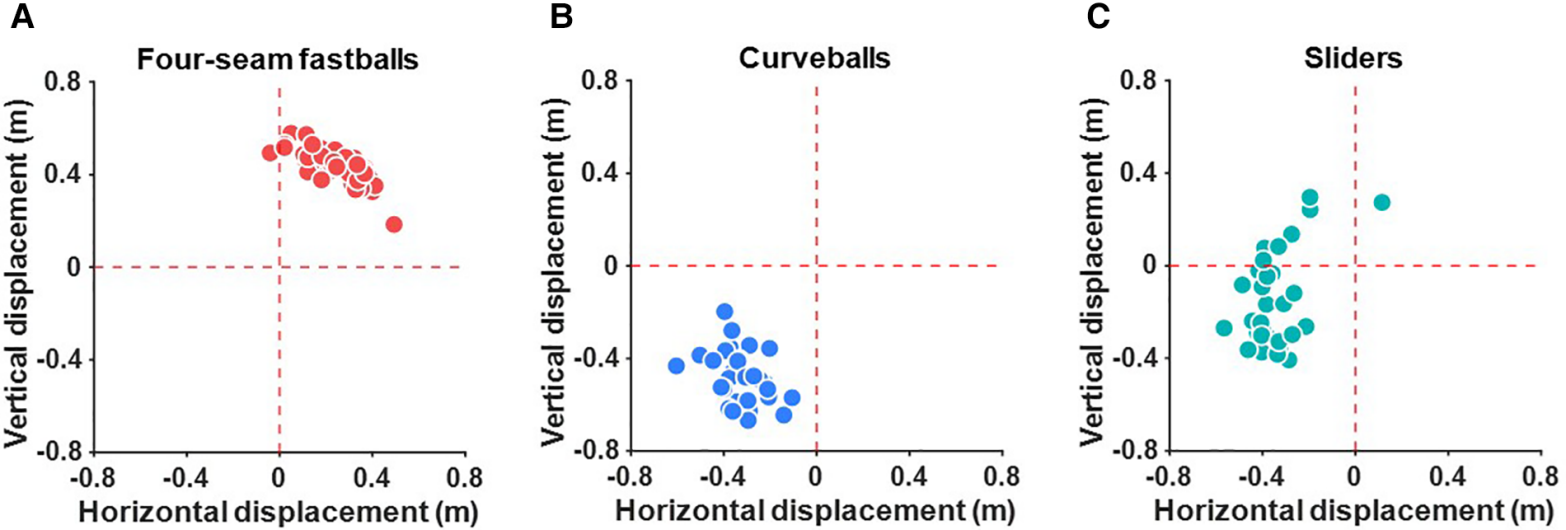
The deviation of (**A**) four-seam fastballs, (**B**) curveballs, and (**C**) sliders.

The deviation of the four-seam fastballs was generally positive in the horizontal and vertical directions (Figure 3A). This shows that the four-seam fastballs involve deviations in the third base and the upward direction. On the other hand, the deviation of the curveballs had a negative value in the horizontal and vertical directions (Figure 3B). These results show that the curveballs involve deviations in the first base and the downward direction. The deviation of the sliders was generally negative in the horizontal direction, whereas it was positive or negative in the vertical direction (Figure 3C).

**Figure 4:**
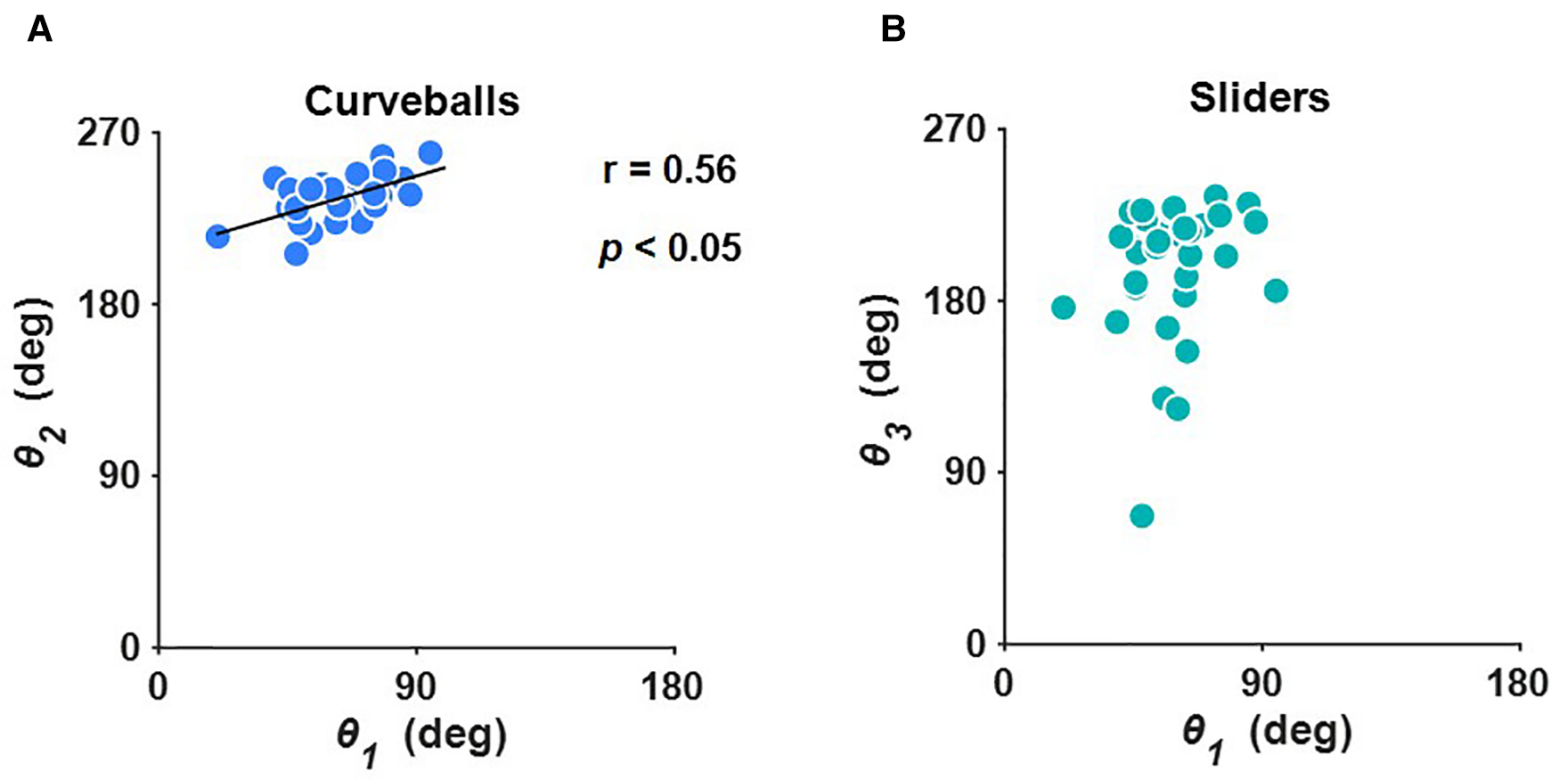
Relationship between (**A**) the angle of four-seam fastballs (*θ_1_*) and curveballs (*θ_2_*), and (**B**) the angle of four-seam fastballs (*θ_1_*) and sliders (*θ_3_*). *r* and *p* stand for the correlation coefficient and probability value, respectively.

We examined whether there was a relationship between the ratio of vertical to left-right deviation in four-seam fastballs and breaking balls and found a statistically significant correlation between the angle of four-seam fastballs (*θ_1_*) and curveballs (*θ_2_*) (*r* = 0.56, *p* < 0.05) (Figure 4A). However, there was no statistically significant correlation between the angle of the four-seam fastballs (*θ_1_*) and sliders (*θ_3_*) (Figure 4B).

**Figure 5:**
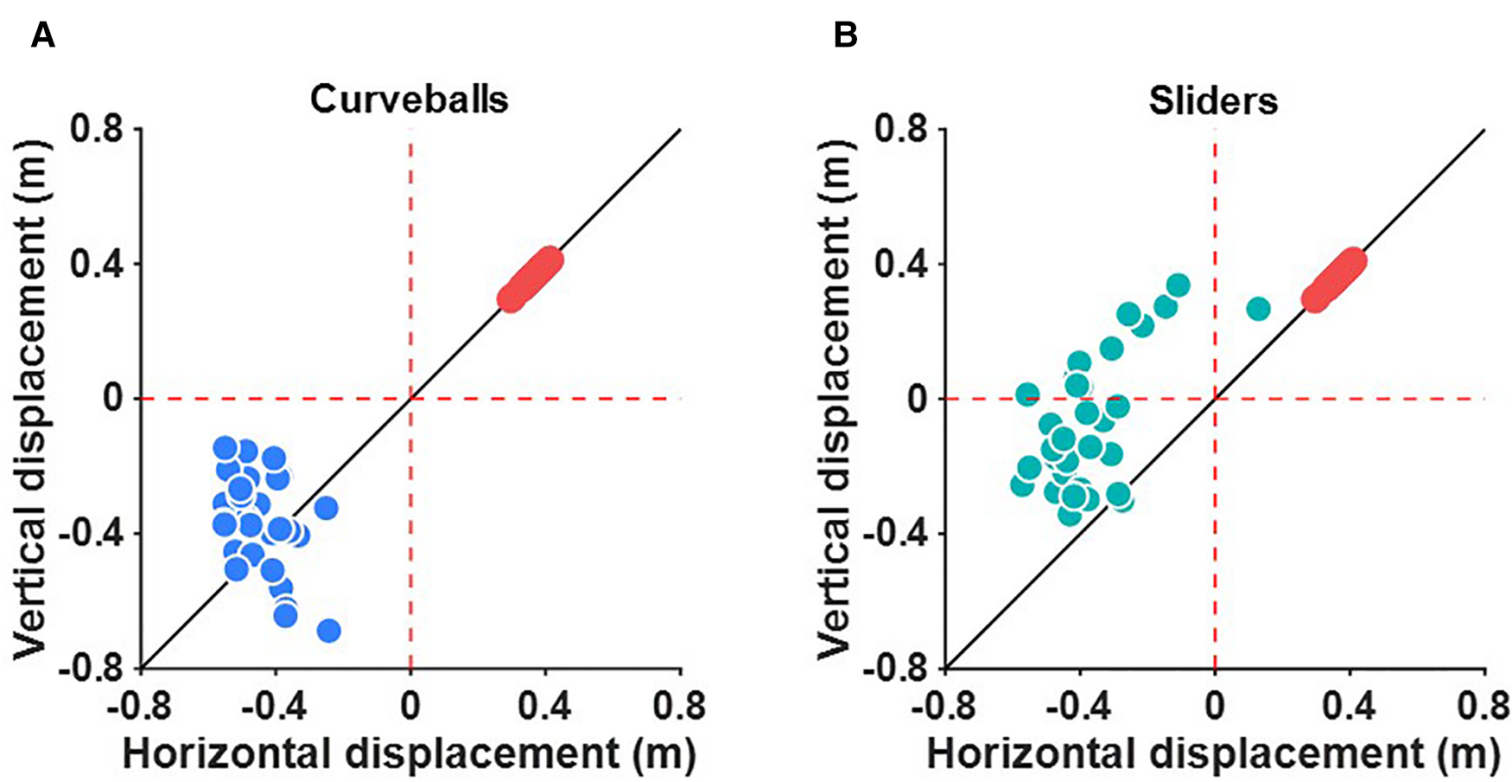
The relative deviation of (**A**) the curveballs and (**B**) the sliders from the four-seam fastballs. The black line indicates that the amount of deviation of the vertical and left-right directions are equal. Red, blue, and green dots indicate the deviation of the four-seam fastballs, the curveballs, and the sliders after conversion.

Figure 5 shows the deviation of the breaking balls from the four-seam fastballs. The deviation of the curveballs from the four-seam fastballs is not always located above the line connecting the origin and the deviation of the four-seam fastballs (Figure 5A). In contrast, the deviations of the sliders from the four-seam fastballs were consistently located above the line connecting the origin and the deviation of the four-seam fastballs (Figure 5B).

## 4. Discussion

The purpose of this study was to characterize the deviation of breaking balls from that of four-seam fastballs. We found that the direction of the deviation of the curveballs can generally be predicted from that of the four-seam fastballs. We also found that the limits of the deviation of the sliders can be determined from the direction of the deviation of the four-seam fastball. These findings clearly show the differences in the characteristics of the curveballs and sliders.

### 4.1. Characteristics of the deviation of the breaking balls from the four-seam fastballs

The deviation of the arrival position for each pitch was determined by comparing the actual trajectory to a free-fall trajectory without considering rotation. Our results showed that the four-seam fastballs deviated to the third base and upward direction, while the curveballs deviated to the first base and downward direction (Figures 3A,B). These results are generally explained by the fact that the four-seam fastballs had a backspin and negative sidespin values, while the curveballs had a topspin and positive sidespin values (Table 1). Our results regarding the direction of the deviations of the four-seam fastballs and curveballs are consistent with those of previous studies (5–7). Sliders deviated upward or downward direction while deviating in the first base direction (Figure 3C). The result was explained by the fact that the sliders generally had a topspin but sometimes a backspin, while sidespin for sliders had positive values (Table 1). Although few studies have quantified the deviation of the arrival position of sliders, our results regarding the direction of the deviation of sliders are consistent with those of Nagami et al. (6). These consistencies support the validity of our results, and we will analyze the deviation in detail.

Determining the angle of each pitch allows us to characterize the ratio of the vertical to left-right deviation. We showed that there was a statistically significant correlation between the angles of the four-seam fastballs (*θ_1_*) and curveballs (*θ_2_*) (Figure 4A). This indicates that the pitcher who throws the four-seam fastballs with a small left-right and large upward deviation tends to throw the curveballs with a small left-right and large downward deviation. It should be noted here that, at 0.56, the correlation coefficient between *θ_1_* and *θ_2_* is moderately weak; thus, it is not possible to strictly predict *θ_2_* from *θ_1_*. For sliders, there was no statistically significant correlation between the angle of the four-seam fastballs (*θ_1_*) and sliders (*θ_3_*) (Figure 4B). In summary, the deviation of the direction of the curveballs can generally be predicted from that of the four-seam fastballs. Conversely, it is difficult to predict the deviation of the direction of the sliders from that of the four-seam fastballs.

While the deviations of the four-seam fastballs were almost within a single quadrant (Figure 3A), the deviations of the sliders varied across multiple quadrants (Figure 3C). As a result, there was no significant correlation between the *θ_1_* and *θ_3_*. To describe the characteristics of the sliders, we calculated the relative deviation of the sliders from the four-seam fastballs. The results of this analysis showed that the relative deviation of the sliders was consistently located above the line connecting the origin and the deviation of the four-seam fastballs (Figure 5B). This indicates that the limits of the deviation of the sliders can be determined from the direction of the deviation of the four-seam fastball. For example, the slider will not deviate more than 0.4 m in a downward direction if the four-seam fastball deviates 0.4 m in the upward and right direction, and the slider deviates 0.4 m in the left direction.

We characterized the difference between the curveballs and sliders. The angle of the four-seam fastballs (*θ_1_*) was correlated with the angle of the curveballs (*θ_2_*), indicating that the direction of the deviation of the curveballs can generally be predicted from that of the four-seam fastballs. For sliders, the limits of the deviation of the sliders can be determined from the direction of the deviation of the four-seam fastball. Most previous studies have described the physical properties of each pitch type (orientation of the translational and angular velocity, speed, spin rate, etc.) (4–7, 14). Our study moved forward with the description of the physical properties of each pitch type which enabled us to extract valuable insights. We hope that examining curveballs and sliders, as well as other pitch types, will provide interesting information.

### 4.2. Limitations

This study had some limitations. First, only one pitch was analyzed for each pitch type. This leads to the possibility that pitched balls with unusual trajectories were analyzed in this study. However, the pitch analyzed in this study was satisfactory, and therefore it is acceptable to assume that a usual trajectory was analyzed.

Second, it was assumed that the ball velocity in the travel direction (***v_y_0_***) was set to the value obtained from the speed gun, and the ball velocity in the left-right (***v_x_0_***) and vertical direction (***v_z_0_***) was 0. However, the actual ball velocity most likely includes some left/right and vertical directions. Because of the assumption that the ball velocity in the left-right (***v_x_0_***) and vertical direction (***v_z_0_***) was 0, a different trajectory of the ball from the actual trajectory was estimated. We examined the validity of this assumption and obtained data on velocity vectors of the four-seam fastballs and breaking balls for the 12 participants not included in the present study. Experiments to obtain this data were conducted in an environment similar to the present study. We used this data to examine the validity of the assumption and confirmed that the assumption did not affect our conclusions.

Third, the mathematical model used in this study to estimate the ball arrival position does not consider the surface material and seam orientation of the ball. The differences in the surface material and seam orientation of the ball affect the ball arrival position. Therefore, the actual ball arrival position and estimated ball arrival position may be different, which has effects on the findings of this study.

### 4.3. Practical implications

Our results and the previous study provide valuable implications to consider when improving the pitching form (15). Jinji et al. (15) showed the direction of the ball spin axis is parallel to the plane of the palm immediately before the ball is released in a four-seam fastball. This implies that a four-seam fastball pitched by a sidearm pitcher has a predominant side spin, and the pitched ball tends to deviate from the left/right directions. Similarly, a four-seam fastball pitched by an overthrow pitcher has a predominant back spin, and the pitched ball tends to deviate vertically. These findings suggest that changes in the pitching form affect the deviation of four-seam fastballs. The ratio of vertical to left-right deviation in the four-seam fastballs correlated with that of the curveballs, according to this study. The findings of this study and the previous study suggest that changes in pitching form affect not only the deviation of the four-seam fastballs but also that of the curveballs. Coaches and players will need to consider this suggestion when improving the pitching form.

Lastly, our results have practical implications from the batter's perspective. Baseball batting is recognized as a sports movement with extremely severe time constraints. This is because the batter should decide to swing approximately 130 ms after the pitcher throws the ball, which is close to the limit of the human visuomotor capacity (16). Despite such time constraints, professional batters can make clean contact with the ball. Therefore, it is believed that the batter predicts the trajectory of the pitched ball (17, 18). In fact, professional batters can accurately predict the trajectory of a pitched ball (19, 20). One way to overcome such professional batters is to not allow them to predict the trajectory of the pitches. If our results are not applicable at the professional level, professional pitchers would throw curveballs and sliders which are difficult for batters to estimate. This may have led professional pitchers to overcome the batters. Conversely, if our results are also true for the professional level, the batter can estimate the deviation of the curveballs and sliders from that of the four-seam fastballs. This makes it easier for the batter to predict the ball's trajectory. In this case, how does a professional pitcher overcome the batters? It would be interesting to examine whether our results are like a strong law obtained at professional and amateur levels, or like a weak law obtained only at an amateur level.

## 5. Conclusion

We found that the ratio of the vertical to left-right deviation in the curveballs correlated with that of the four-seam fastballs, indicating that the direction of the deviation of the curveballs can generally be predicted from that of the four-seam fastballs. We also found that the limits of the deviation of the sliders can be determined from the direction of the deviation of the four-seam fastball. This study allowed us to predict the deviation of the breaking ball from the four-seam fastballs and clearly showed the differences in the characteristics between curveballs and sliders.

## Data Availability

The raw data supporting the conclusions of this article will be made available by the authors, without undue reservation.
